# Trauma histories among justice-involved youth: findings from the National Child Traumatic Stress Network

**DOI:** 10.3402/ejpt.v4i0.20274

**Published:** 2013-07-16

**Authors:** Carly B. Dierkhising, Susan J. Ko, Briana Woods-Jaeger, Ernestine C. Briggs, Robert Lee, Robert S. Pynoos

**Affiliations:** 1 National Center for Child Traumatic Stress, University of California, Los Angeles, CA, USA; 2 National Center for Child Traumatic Stress, Duke University School of Medicine, Durham, NC, USA

**Keywords:** Juvenile justice, trauma, post-traumatic stress, delinquency, mental health, age of onset, adolescent, NCTSN

## Abstract

**Background:**

Up to 90% of justice-involved youth report exposure to some type of traumatic event. On average, 70% of youth meet criteria for a mental health disorder with approximately 30% of youth meeting criteria for post-traumatic stress disorder (PTSD). Justice-involved youth are also at risk for substance use and academic problems, and child welfare involvement. Yet, less is known about the details of their trauma histories, and associations among trauma details, mental health problems, and associated risk factors.

**Objective:**

This study describes detailed trauma histories, mental health problems, and associated risk factors (i.e., academic problems, substance/alcohol use, and concurrent child welfare involvement) among adolescents with recent involvement in the juvenile justice system.

**Method:**

The National Child Traumatic Stress Network Core Data Set (NCTSN-CDS) is used to address these aims, among which 658 adolescents report recent involvement in the juvenile justice system as indexed by being detained or under community supervision by the juvenile court.

**Results:**

Age of onset of trauma exposure was within the first 5 years of life for 62% of youth and approximately one-third of youth report exposure to multiple or co-occurring trauma types each year into adolescence. Mental health problems are prevalent with 23.6% of youth meeting criteria for PTSD, 66.1% in the clinical range for externalizing problems, and 45.5% in the clinical range for internalizing problems. Early age of onset of trauma exposure was differentially associated with mental health problems and related risk factors among males and females.

**Conclusions:**

The results indicate that justice-involved youth report high rates of trauma exposure and that this trauma typically begins early in life, is often in multiple contexts, and persists over time. Findings provide support for establishing trauma-informed juvenile justice systems that can respond to the needs of traumatized youth.

Youth involved in the juvenile justice system report higher rates of trauma exposure, post-traumatic stress disorder (PTSD), and other mental health problems (e.g., depression, anxiety) compared to the general population (Schufelt & Cocozza, 2006; Wolpaw & Ford, 2004; Wood, Foy, Layne, Pynoos, & James, 2002). Justice-involved youth also tend to experience multiple types of trauma, or polyvictimization, before they reach the juvenile justice system (Abram et al., 2004). Yet, less is known about the details of their trauma histories such as prevalence rates of a broad range of trauma types, rates of co-occurring trauma across childhood, and the age of onset of trauma exposure.

## Trauma exposure and PTSD among justice-involved youth

The relation between trauma exposure and juvenile justice involvement has been consistently documented (Chamberlain & Moore, 2002; Ford, Chapman, Hawke, & Alpert, 2007; Kerig & Becker, 2010; Widom & Maxfield, 1996). Youth who report child maltreatment, both through official case records or self-reports, are found to be at higher risk for delinquent or criminal involvement in both adolescence and adulthood (Smith & Thornberry, 1995; Widom & Maxfield, 1996). In addition, more severe forms of maltreatment (i.e., chronic or frequent maltreatment) have been found to be associated with more severe and chronic delinquent behavior and the relation between child maltreatment and justice involvement holds across gender and ethnicity (Smith & Thornberry, 1995; Widom & Maxfield, 1996). Other forms of trauma exposure, beyond child maltreatment, have also been linked to delinquency and justice involvement, such as community violence, domestic violence, and traumatic loss (Foy, Ritchie, & Conway, 2012; Kerig, Ward, Vanderzee, & Moeddel, 2009; Wood et al., 2002).

Prevalence rates of trauma exposure among youth involved in the juvenile justice system highlight this robust relation. One study found 92% of justice-involved youth reported exposure to at least one type of trauma, and that exposure to multiple traumas was the norm (Abram et al., 2004). Females tend to report higher rates of interpersonal victimization, particularly sexual assault, while males report higher rates of witnessing violence (Cauffman, Feldman, Waterman, & Steiner, 1998; Ford et al., 2007; Foy et al., 2012). For instance, 29% of incarcerated females compared to 3% of their incarcerated male counterparts reported being raped or molested (Wood et al., 2002), and 48% of incarcerated males compared to 17% of incarcerated females reported witnessing some type of violent act (Cauffman et al., 1998).

In light of the high rates of trauma exposure among justice-involved youth, many prevalence studies have focused specifically on the development of PTSD among this population. Rates of PTSD tend to vary between 3 and 50% among incarcerated youth (Ford et al., 2007) with a 30% prevalence rate on average. For example, a study comparing 96 females and 93 males incarcerated in the California Youth Authority found that nearly half of the females (49%) met the criteria for PTSD compared to about one-third (32%) of males (Cauffman et al., 1998). Another study of randomly selected youth (*N*=898) in a pre-trial detention center in Cook County, Illinois found about 11% of males and 15% of females met the criteria for PTSD (Abram et al., 2004). The discrepancies among prevalence rates are attributed to regional differences among study participants, the use of varying assessment instruments, and the time at which the assessment occurs during juvenile justice processing (Wolpaw & Ford, 2004).

While trauma exposure and PTSD are common among justice-involved youth, it is not yet clear what the mechanisms of influence are between trauma and delinquency (Ardino, 2012; Kerig, 2012a). The few studies that have begun to illuminate this process focus on emotional and cognitive processes as mediating mechanisms (Allwood, Baetz, DeMarco, & Bell, 2012; Allwood & Bell, 2008; Kerig & Becker, 2010). For instance, post-traumatic stress symptoms and cognitions supportive of violence have been found to mediate the relation between violence exposure (i.e., family and community violence exposure) and self-reported delinquency among a community sample of adolescents (Allwood & Bell, 2008). Post-traumatic stress symptoms have also been found to mediate the relation between violence exposure and additional mental health problems among an incarcerated sample of adolescents (Kerig & Becker, 2012). Importantly, gender differences are consistently found when delineating the relation between trauma and delinquency indicating varying trajectories from trauma to delinquency for males and females (Kerig & Becker, 2012).

An understudied aspect in the developmental trajectory of trauma and delinquency is the age of onset of trauma in this population. This is surprising given the extensive literature on the age of onset of delinquent behavior; one of the most robust predictors of chronic and persistent delinquency (Natsuaki, Ge, & Wenk, 2008; Sampson & Laub, 1993). This literature indicates that the experience of risk factors (e.g., parenting problems, conduct problems, academic failure, peer rejection) early in life is associated with more chronic delinquency and that children who begin their delinquent careers in childhood, rather than later in adolescence, become the most consistent and chronic offenders (Moffitt, 1993; Patterson, DeBaryshe, & Ramsey, 1989). Given the importance of timing in the development of delinquent behaviors, it follows that timing of trauma may also be related to adverse outcomes. The timing of a traumatic experience is also important given that youth who experience trauma early in life are more likely to experience other types of trauma later in life (Finkelhor, Ormrod, & Turner, 2007) and the experience of multiple trauma types is associated with increased post-traumatic stress reactions, difficulties in emotion regulation, and internalizing problems (Finkelhor, Turner, Hamby, & Ormrod, 2011). However, these associations have not been explored among justice-involved samples. Expanding our knowledge regarding the age of onset of trauma exposure can enhance our understanding of the developmental implications of trauma exposure and justice involvement.

## Mental health and associated risk factors among justice-involved youth

Justice-involved youth often experience additional adversity and mental health problems, beyond trauma exposure and PTSD, either preceding or concurrent with justice involvement. In a nationally representative study, approximately 70% of justice-involved youth met criteria for at least one mental health disorder, and among those youth 79% met criteria for two or more diagnoses (Schufelt & Cocozza, 2006). The most common disorders include disruptive disorders, substance use disorders, anxiety disorders, and mood disorders. PTSD and other mental health problems tend to co-occur among highly traumatized samples as well. For instance, in the National Survey of Adolescents, Ford and colleagues (2010) found that adolescents exposed to multiple trauma types compared to non-exposed adolescents had double the risk for major depressive disorder, triple the risk for PTSD, and 5–8 times the risk for comorbid disorders (Ford, Elhai, Connor, & Frueh, 2010).

Substance-use problems, academic problems, and concurrent child welfare involvement are also common among justice-involved youth. For instance, 1.9 million of the 2.4 million youth arrested in 2000 reported a substance-abuse problem, were arrested for a drug-related offense, and/or were under the influence at the time of their arrest (National Center on Addiction and Substance Abuse, 2004). Poor academic performance is associated with increased delinquent involvement (Maguin & Loeber, 1996), and many youth drop out of school after release from a juvenile justice facility (Buffington, Dierkhising, & Marsh, 2010). Additionally, up to 42% of youth in the juvenile justice system are crossover youth, youth who report involvement in both the juvenile justice and child welfare systems, with females representing a higher proportion of crossover youth (Herz & Ryan, 2008; Herz, Ryan, & Bilchik, 2010). While these risk factors are thought to contribute to and/or co-occur with justice involvement, they are often associated with PTSD and trauma exposure also. However, less is known about the associations among these risk factors in justice-involved samples. A better understanding of these associations can improve intervention and prevention efforts for youth.

## The current study

This study describes detailed trauma histories, mental health problems, and associated risk factors (i.e., academic problems, substance/alcohol use, and concurrent child welfare involvement) among adolescents with recent involvement in the juvenile justice system. Justice-involved youth include 658 adolescents (aged 13–18 years) from the National Child Traumatic Stress Network Core Data Set (NCTSN-CDS) who report recent involvement in the juvenile justice system as indexed by being detained or under community supervision by the juvenile court. Four primary questions guide this descriptive study: (1) What are the prevalence rates of trauma types, mental health problems, and associated risk factors (i.e., academic problems, substance/alcohol use, and concurrent child welfare involvement) among justice-involved youth?; (2) Are there gender differences in trauma types, mental health problems, and associated risk factors?; (3) At what age are youth first experiencing trauma and does trauma co-occur (i.e., multiple trauma types occurring within a single year)?; and (4) How is age of onset of trauma associated with mental health problems and related risk factors among males and females?

## Method

### Participants

The National Child Traumatic Stress Network (NCTSN) is a federally funded initiative that seeks to raise the standard of care and increase access to services for traumatized children and their families. As part of this initiative, the Core Data Set (CDS) was established to standardize assessment protocols across all funded NCTSN clinical sites. These sites included a range of community-based mental health clinics, child welfare settings, juvenile justice programs, hospitals, schools, and residential treatment centers. Data were collected between 2004 and 2010, from 56 sites located across the country and includes baseline assessments and follow-up treatment information and outcomes. All participants (*N*=14,088 children and adolescents from birth to 21 years) were referred for trauma-focused treatment and assessed on various clinical measures, such as mental health problems, functional impairment, treatment types, and service system utilization. Extensive training on assessment administration and data entry was provided to all participating sites. A clinical service provider working with the referred youth and their parents/caregivers completed all assessment instruments. Only baseline assessments were used for this study.

The justice-involved subgroup (*n*=658) includes adolescents aged 13–18 years who indicated recent involvement with the juvenile justice system as defined by either: (1) being in a detention center, training school, jail, or prison (14.6%); (2) having seen a probation officer or court counselor (57.9%); or (3) both (27.5%) within the past 30 days. The sample is racially and ethnically diverse with 40.1% identifying as White, 21.6% identifying as Black, 31.4% identifying as Hispanic, and 6.9% identifying as Other. The sample is composed of more females (54%) than males (46%) and the average age is 15.7 years (*SD*=1.3). The majority of the sample lives at home with their parents (53.6%), with 23.9% in either a correctional facility or residential treatment center, 8.4% with other family members, 6.9% in foster care, and 7.2% in another living situation (i.e., homeless, independent, or other). Approximately two-thirds (67.5%) of the sample reported eligibility for public insurance.

### Instruments

#### Trauma exposure

The trauma history profile (THP) is a comprehensive assessment of an individual's trauma history including type of trauma and when it occurred in the life span. The THP includes information regarding age of onset and whether more than one trauma type co-occurred in the same year. The THP is derived from the trauma history component of the UCLA PTSD-Reaction Index (PTSD-RI: Steinberg, Brymer, Decker, & Pynoos, 2004) and expanded to include 19 trauma types. The provider at intake or early in the course of service delivery completed it. Trauma history information is obtained retrospectively from multiple informants, including the adolescent, parents/caregivers, and/or other relatives. Definitions for many of the trauma types were adapted from the National Child Abuse and Neglect Data System (NCANDS) Glossary, a national database of child abuse and neglect reports.

#### Post-traumatic stress reactions

The UCLA PTSD-RI was used to capture the frequency of post-traumatic stress symptoms over the past month, with response options ranging from 0 (none of the time) to 4 (most of the time). Scoring algorithms permit tabulation of a PTSD-RI total score, as well as Criterion B, C, and D symptom subscale scores. For this study, a total PTSD score is a summed continuous variable created from the symptom items that correspond to diagnostic criteria as defined by the Diagnostic and Statistical Manual for Mental Health Disorders (DSM-IV-TR: American Psychiatric Association [APA], 2000). A clinical cut-off of 38 is then used to categorize those in the clinical range (i.e., most likely to meet criteria for PTSD) as described by Steinberg and colleagues (2004). Clinically significant symptom cluster scores (i.e., Criterion B–D) are derived from whether or not a specific number of symptoms were present in each cluster based on the DSM-IV-TR criteria. For Criterion B, the DSM-IV-TR requires the presence of at least one symptom in the past month, for Criterion C at least three symptoms, and for Criterion D at least two symptoms. A symptom is considered “present” when the respondent indicates the symptom occurred much of the time (2–3 times a week in the past month) or most of the time (almost everyday in the past month). Psychometric properties are fairly robust with good to excellent internal reliability across age, racial/ethnic groups, and gender (Steinberg et al., 2004Steinberg et al., 2013).

#### Internalizing and externalizing problems

The Child Behavior Checklist (CBCL; Achenbach & Rescorla, 2001) was used to assess internalizing and externalizing symptoms. The CBCL is completed by a parent or caregiver who knows the child well. This widely used measure consists of 118 items scored on a 3-point scale ranging from 0 (not true) to 2 (often true) and yields scores on two broad band scales of internalizing and externalizing, as well as scores on DSM-IV-oriented scales, and empirically based syndrome scales that reflect emotional and behavioral problems and symptoms. The measure has been found to have sound psychometric properties with respect to reliability and validity, across racially and ethnically diverse samples.

#### Associated risk factors

To assess for academic problems, and substance/alcohol use, clinicians used a 3-point scale ranging from 0 (not a problem), 1 (somewhat a problem), and 2 (very much a problem) to rate the degree of impairment in youth within the last 30 days. Responses indicating “somewhat a problem” and “very much a problem” were collapsed to create a dichotomous variable. Child welfare involvement was determined when youth indicated that they received services within the last 30 days from the child welfare system (yes/no). For this study, items assessing involvement in foster care, Department of Social Services (DSS), and child welfare were collapsed to create a child welfare involvement variable.

## Results

### What are the prevalence rates of trauma types, mental health problems and associated risk factors (i.e., academic problems, substance/alcohol use, and concurrent child welfare involvement) among justice-involved youth?

**Fig. 1:**
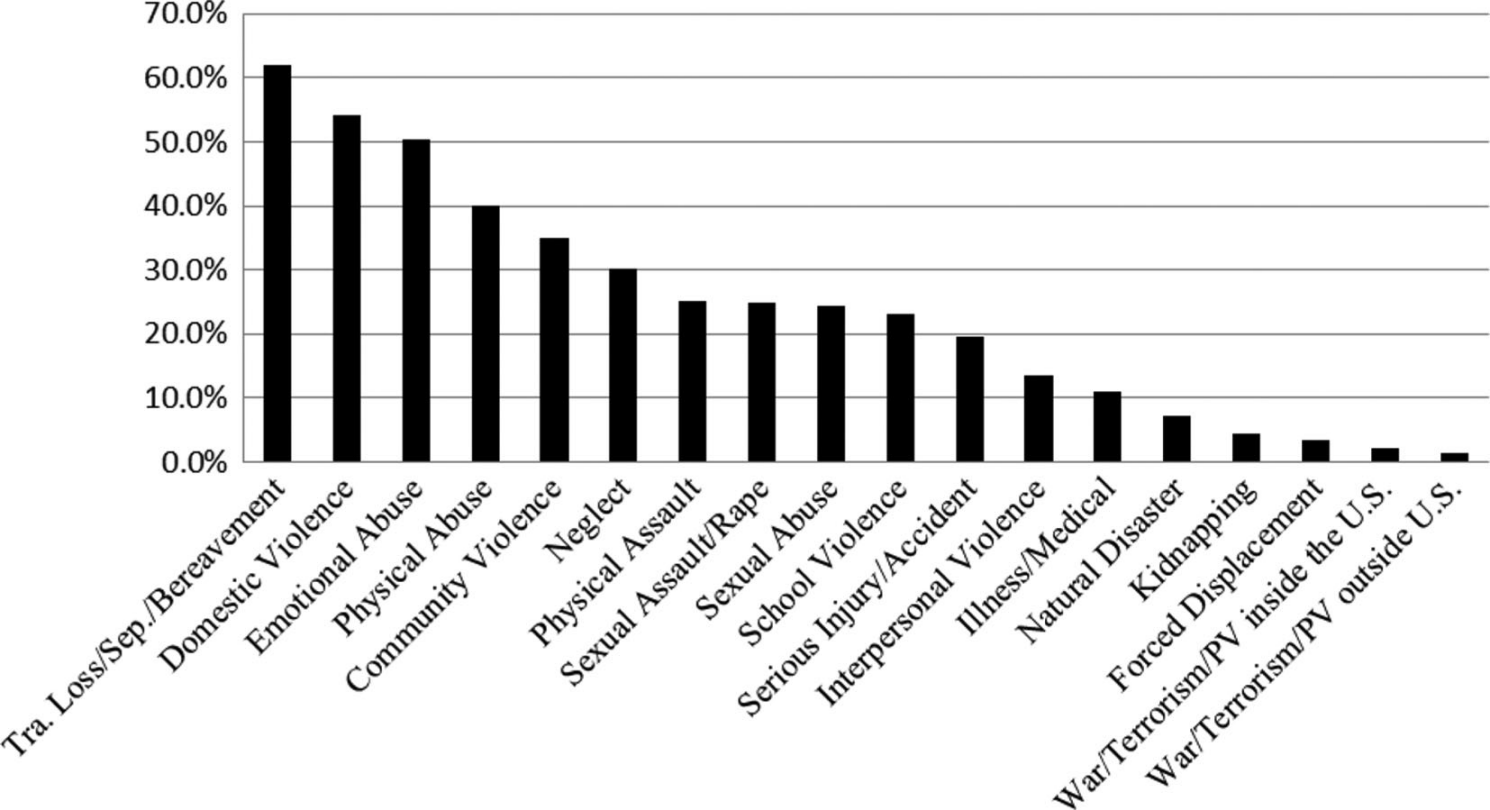
Prevalence rates of trauma exposure by trauma type.

The average number of different trauma types experienced among adolescents in the sample is 4.9 (*SD*=2.9). As shown in Fig. 1, the most frequently reported trauma types are loss and bereavement (i.e., traumatic loss, separation from caregiver, or bereavement) (61.2%), impaired caregiver (51.7%), domestic violence (51.6%), emotional abuse/psychological maltreatment (49.4%), physical maltreatment/abuse (38.6%), and community violence (34%).

**Fig. 2:**
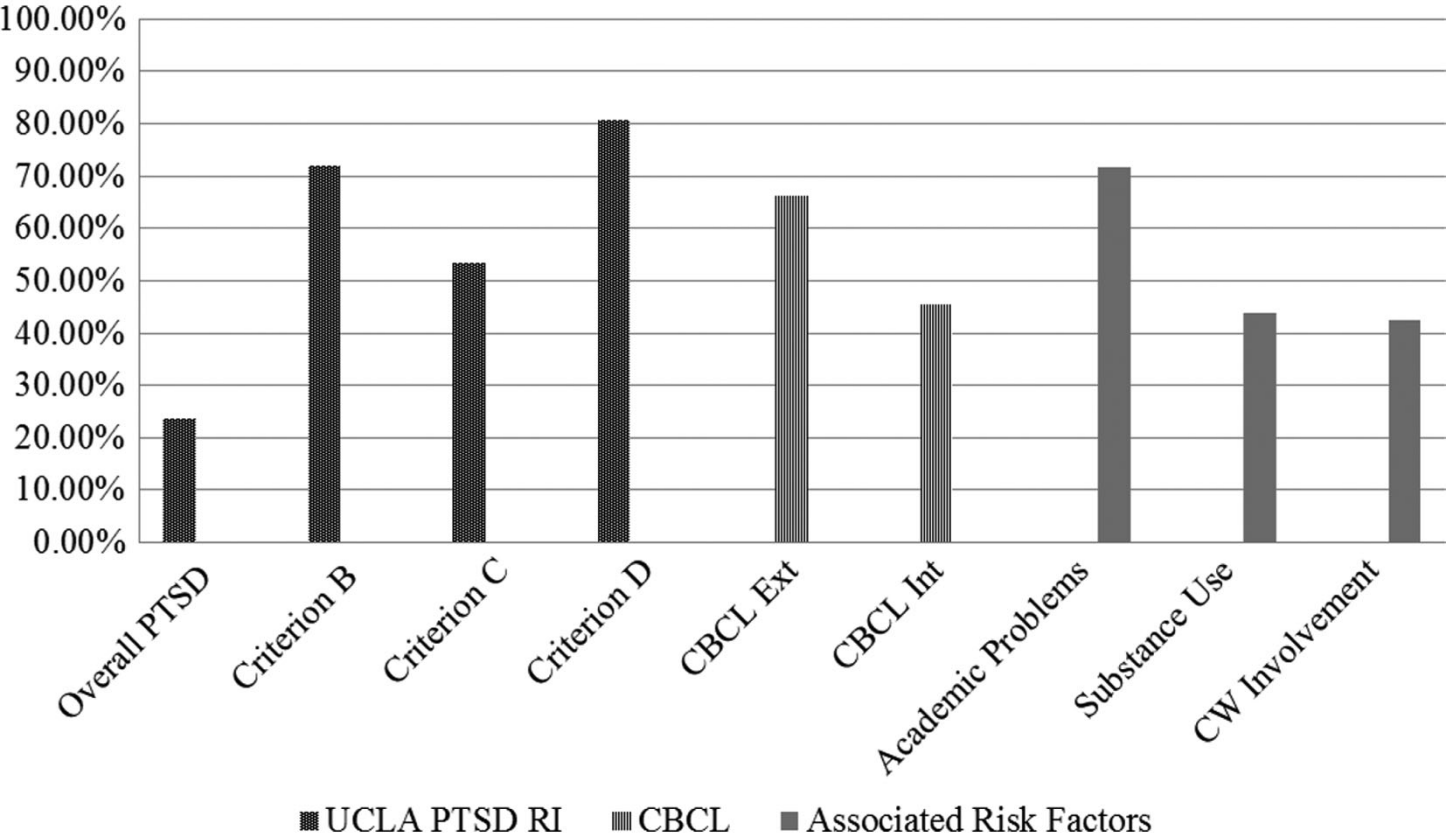
Percent of youth in the clinical range for mental health problems and prevalence rates of associated risk factors.

As shown in Fig. 2, adolescents reported high levels of post-traumatic stress symptoms with 23.6% in the clinical range for PTSD. Additionally, the majority of adolescents reached the clinical range on specific PTSD symptom clusters (i.e., re-experiencing, hyperarousal, avoidance). For Criterion B, 71.8% of the sample was in the clinical range, 53.2% for Criterion C symptoms and 80.6% for Criterion D symptoms. The majority of the sample (66.1%) reported externalizing problems in the clinical range and nearly half (45.5%) reported internalizing problems in the clinical range. Within the externalizing domain, rule breaking (37%) and aggressive behavior (34.1%) were the most frequently endorsed behaviors, followed by attention problems (20.1%) and social problems (15.5%). Internalizing symptoms were more evenly split with 22.3% endorsing withdrawn/depressed symptoms and thought problems, 21% endorsing anxious/depressed symptoms, and 20.1% endorsing somatic complaints. Adolescents also reported substantial academic problems (71.8%), substance/alcohol use (43.8%), and concurrent child welfare involvement (42.2%).

### Are there gender differences in trauma types, mental health problems, and associated risk factors?

Differences in mental health problems and associated risk factors between genders were assessed using Type 3 tests from mixed general linear models for continuous variables and mixed logistic models for binary variables. A classic Bonferroni correction was then used which required *p*-values ≤0.005 for significance (Rosenthal & Rosnow, 2007). As shown in Table 1, both males and females showed relatively similar rates of exposure to each type of trauma with the exception of sexual abuse and assault where females had higher rates. However, females reported significantly higher rates of total PTSD (*F*(1,24)=13.17, *p* <0.005), Criterion B symptoms (*F*(1,24)=18.00, *p* <0.005), and concurrent child welfare involvement (*F*(1,23)=14.29, *p* <0.005). There were no significant differences between internalizing and externalizing problems, substance/alcohol use, and academic problems among males and females at the 0.005 significance level.

**Table 1:** Prevalence^1^ of trauma types by ender

	Male	Female
	
Trauma type^2^	*N*=303	*N*=355
Sexual maltreatment/abuse	45 (15.5%)	109 (31.8%)
Sexual assault/rape	26 (8.8%)	130 (38.7%)
Physical maltreatment/abuse	115 (39%)	139 (40.6%)
Physical assault	77 (26.6%)	83 (24.1%)
Emotional abuse/psychological maltreatment	137 (46.3%)	188 (53.9%)
Neglect	90 (30.7%)	102 (29.7%)
Domestic violence	147 (51.4%)	193 (56.3%)
War/terrorism/PV inside the United States	3 (1%)	11 (3.1%)
War/Terrorism/PV outside United States	6 (2%)	4 (1.1%)
Illness/medical	34 (11.3%)	38 (10.9%)
Serious injury/accident	60 (20.1%)	67 (19%)
Natural disaster	21 (7%)	26 (7.4%)
Kidnapping	10 (3.3%)	19 (5.4%)
Traumatic loss or bereavement	174 (58.6%)	229 (64.9%)
Forced displacement	5 (1.7%)	17 (4.8%)
Impaired caregiver	140 (47.5%)	200 (57.3%)
Extreme interpersonal violence	38 (12.9%)	50 (14.3%)
Community violence	119 (40.8%)	105 (30.1%)
School violence	67 (23%)	80 (23.1%)

1 Percentage based on entire relevant population, not excluding “missing” for each trauma type.

2 Trauma types are not mutually exclusive.

### At what age are youth first experiencing trauma and does trauma co-occur (i.e., multiple trauma types occurring within a single year)?

**Fig. 3:**
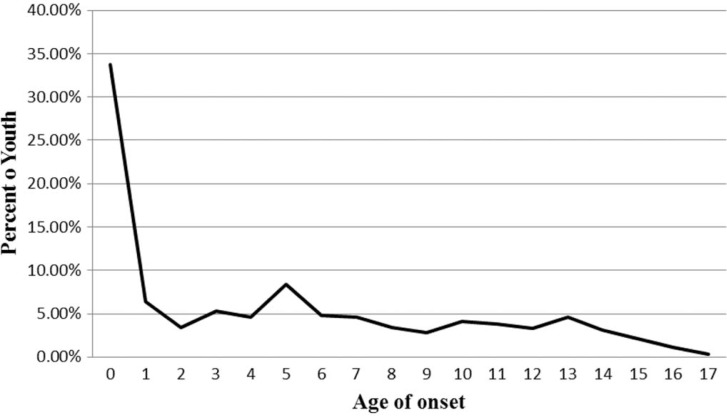
Distribution of age of first trauma exposure.

**Fig. 4:**
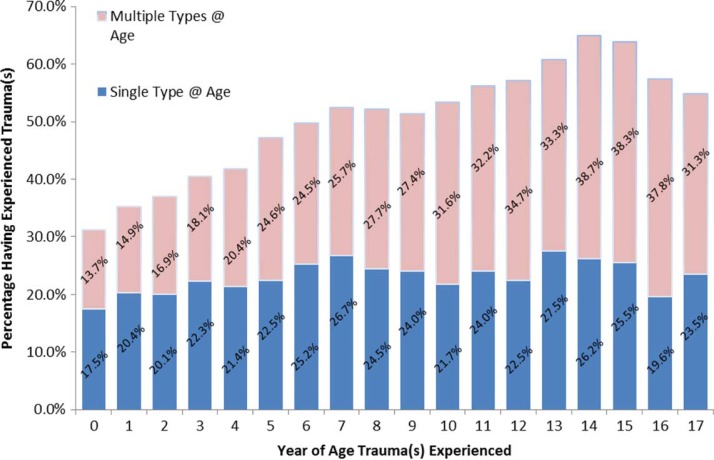
Prevalence rates of multiple types and single type of trauma exposure averaged each year by age.

Age at first trauma exposure was overwhelmingly early in the youth's lives. As shown in Fig. 3, age of onset for trauma exposure occurred within the *first year* of life for 33.72% of youth, followed by 28.42% of youth who first experienced trauma in years one through five. Thus, more than half (62.14%) of the group experienced trauma in the first 5 years of life. While trauma first occurred in early childhood, the prevalence rate of exposure to co-occurring trauma generally increased at each age from childhood into adolescence (Fig. 4). By age 5, one-quarter to one-third of youth report co-occurring trauma exposure at each age. The majority of youth (90%) experienced multiple trauma types and only 10% experienced a single trauma type at the time of assessment, regardless of frequency or duration of exposure.

### How is age of onset of trauma associated with mental health problems and associated risk factors among males and females?

As shown in Table 2, there are differences between males and females in the associations among age of onset and mental health problems. For females, early age of onset was associated with higher total PTSD (*r*=−0.148, *p*=0.01), Criterion C (*r*=−0.139, *p*=0.015), and Criterion D symptoms (*r*=−0.158, *p*=0.006) but not for males. Early age of onset was associated with both externalizing and internalizing problems for males (*r*=−.0.332, *p* <0.01; *r=*−0.233, *p* <0.01) and females (*r*=−0.153, *p* <0.05; *r*=−0.175, *p* <0.01), respectively; though the magnitudes of the correlations were larger for males. Age of onset was highly correlated to exposure to multiple trauma types for both males (*r*=−0.406, *p* <0.001) and females (*r=*−0.404, *p* <0.001). Finally, age of onset was related to child welfare involvement for males (*r=*−0.152, *p* <0.05) and females (*r*=−0.146, *p* <0.01), but not with academic problems or substance/alcohol use.

**Table 2:** Correlations between selected variables, females in upper brackets and males in lower brackets

Measure	1	2	3	4	5	6	7	8	9	10	11	12
1. Age^1^	–	0.03	0.21***	0.01	0.07	0.09	0.07	0.12	−0.04	−0.22***	−0.04	0.08
2. Age/first exposure^2^	0.09	–	−0.40***	−0.10	−0.14^*^	−0.16**	−0.15*	−0.18**	−0.15*	−0.09	−0.15**	0.06
3. Number of trauma types	0.08	−0.41***	–	0.20***	0.18***	0.16**	0.21***	0.27***	0.06	−0.05	0.29***	0.01
4. Criterion B	0.07	0.01	0.27***	–	0.70***	0.61***	0.89***	0.30***	0.09	0.14*	−0.06	0.06
5. Criterion C	0.09	−0.01	0.29***	0.70***	–	0.61***	0.91***	0.37***	0.22***	0.16**	−0.01	0.08
6. Criterion D	0.07	−0.08	0.28***	0.57***	0.61***	–	0.81***	0.28***	0.19**	0.14*	−0.00	0.09
7. PTSD total	0.09	−0.03	0.32***	0.87***	0.91***	0.82***	–	0.38***	0.20**	0.17**	−0.03	0.09
8. CBCL – int	−0.02	−0.23**	0.20**	0.20*	0.23**	0.20*	0.25**	–	0.49***	0.13	−0.03	0.08
9. CBCL – ext	−0.09	−0.33**	0.15*	0.08	0.11	0.24**	0.161*	0.55***	–	0.27***	−0.15*	0.34***
10. Academic problems	−0.07	−0.07	0.14*	0.04	0.03	0.06	0.05	0.07	0.19**	–	−0.04	0.11
11. Welfare involvement	−0.13*	−0.15*	0.03	−0.08	−0.16*	−0.10	−0.13*	0.07	0.00	−0.05	–	−0.20***
12. Substance use	0.24***	0.05	0.06	0.14*	0.10	0.17*	0.15*	0.12	0.13	0.12*	−0.10	–

Intercorrelations for female subjects (*n*=355) are presented above the diagonal, and intercorrelations for male subjects (*n*=303) are presented below the diagonal.

1 Age refers to age at baseline evaluation.

2 Age/first exposure refers to the earliest age at which the subject reports trauma experience.

* *p*<0.05

** *p*<0.01

*** *p*<0.001.

## Discussion

### Overview of findings

This study describes the trauma histories, mental health problems, and associated risk factors among adolescents with recent involvement in the juvenile justice system. Mental health problems were prevalent with nearly one-quarter (23.6%) of youth meeting criteria for PTSD. Furthermore, over half of the sample indicated post-traumatic stress symptoms in the clinical range on at least one symptom cluster. Youth overwhelmingly presented with academic problems, substance/alcohol use, and concurrent child welfare involvement. Findings also reveal that youth with recent involvement in the justice system tended to be exposed to trauma beginning early in life and continued to experience multiple types of trauma. Additionally, early age of onset of trauma was associated with exposure to multiple types of trauma for both males and females, while early age of onset was differentially associated with mental health problems among males and females.

### Practice implications

Findings from this study have implications for both practitioners and policymakers. At the practice level, it is clear that screening for trauma exposure, PTSD, and internalizing problems is needed among justice-involved youth. The juvenile justice system is in a unique position to address the multiple problems that impact the lives of justice-involved youth as it has contact with, and often oversight of, this vulnerable population. Beyond screening, clinical assessments are imperative to clearly identify clinical disorders and related functional impairments that guide treatment planning. In light of scarce resources, screening and assessment tools can, and should, be used to direct resources to those most in need.

While PTSD is prevalent among the sample, it was also found that many youth who do not meet criteria for a diagnosis of PTSD are still experiencing clinically significant post-traumatic stress symptoms within individual symptom clusters. For practitioners working with this population, utilizing a conservative cut-off score or methodology when screening for PTSD may more accurately identify youth experiencing clinically significant post-traumatic stress reactions. With this screening method, a follow-up clinical assessment could then be used to evaluate how symptoms may be adversely impacting youth's functioning.

An essential aspect of an effective screening and assessment process is the availability of evidence-based practices for justice-involved youth experiencing trauma reactions. Fortunately, there is an emerging literature on promising practices and evidence-based treatments for justice populations (Kerig, 2012b). Trauma Affect Regulation: Guide for Education and Therapy (TARGET; Ford & Russo, 2006) has been found to reduce disciplinary incidents and punitive sanctions (Ford & Hawke, 2012) and, when compared to treatment as usual, a reduction in mental health problems among incarcerated youth (Marrow, Knudsen, Olafson, & Bucher, 2012). Other interventions have built upon existing evidence-based treatments by adding a trauma-informed approach (Kerig & Alexander, 2012; Smith, Chamberlain, & Deblinger, 2012). For example, an innovative pilot study of an intervention which integrated components of Trauma Focused-Cognitive Behavioral Therapy (TF-CBT; Cohen, Mannarino, & Deblinger, 2006) with Multidimensional Treatment Foster Care (MTFC; Chamberlain, 2003) found a reduction in trauma-related symptoms and delinquency compared to treatment as usual (Smith et al., 2012). Continued intervention studies are needed to further support and disseminate trauma-focused treatment for justice-involved youth.

In light of the prevalence of trauma and post-traumatic stress, staff who have direct and consistent contact with justice-involved youth, such as probation officers and detention staff, should be trained to understand trauma and post-traumatic reactions so they are best equipped to recognize potential emotional distress and post-traumatic stress reactions (Griffin, Germain, & Wilkerson 2012; Marrow et al., 2012). While these staff members are not expected to conduct a clinical assessment (nor are they qualified to), knowledge of trauma and post-traumatic stress can facilitate a better understanding and anticipation of the problems that may arise for justice-involved youth. In addition, trauma-informed training can help staff members who are not clinically trained to make appropriate referrals to mental health practitioners when needed, as they may have the most frequent and direct contact with youth. Indeed, recent research has shown that implementation of a trauma-informed approach using both trauma training for direct care staff and a trauma-focused intervention was effective in reducing psychological distress among youth and improving management of youth problem behaviors (e.g., reductions in seclusions and restraints) when compared to treatment as usual (Marrow et al., 2012).

### Policy implications

It is important for policymakers to acknowledge that justice-involved youth have strikingly high rates of trauma exposure and that this trauma typically begins early in life, is often in multiple contexts (e.g., home, community, school), and persists over time. In light of these findings, prevention and intervention policies should target young children exposed to violence in order to reduce the likelihood of re-victimization and mental health problems, as well as prevent future justice involvement. For youth who do come to the attention of the juvenile court, it is imperative that the system is prepared to meet the needs of chronically traumatized youth with significant mental health problems. Policies that support a trauma-informed juvenile justice system should emphasize trauma screening and assessment, evidence-based trauma treatment, cross-system engagement, and promote resilience and engagement among youth and families (Griffin et al., 2012; Ko & Sprague, 2007).

Attention should also be paid to youth who are not diverted at the point of contact with the juvenile court, resulting in incarceration in a detention or residential treatment facility. These youth are, perhaps, most vulnerable as all other prior interventions have not been successful and they are more likely to recidivate as a juvenile or as an adult, and have poor long-term economic, academic, and mental health outcomes (Justice Policy Institute, 2009; Widom & Maxfield, 1996). Incarceration can be traumatic for youth and abusive practices that are common among large-scale detention facilities may continue to expose youth to trauma and abuse (Mendel, 2011). Policies that promote safety and treatment in these facilities are needed in order to protect and rehabilitate youth in the deepest parts of the juvenile justice system.

These findings also highlight important gender differences among justice-involved youth. We found early age of onset of trauma exposure was significantly correlated with increased post-traumatic stress reactions among females but not males. Additionally, females reported significantly higher rates of post-traumatic stress reactions compared to males. These findings indicate a need for a gender responsive approach. Acknowledging and addressing the distinct needs of males and females is an integral part of juvenile justice reform efforts, while additional research and funding mechanisms to enhance gender responsiveness are needed (Office of Juvenile Justice and Delinquency Prevention [OJJDP], 1998; Watson & Edelman, 2012).

### Limitations and strengths

The current study's findings must be considered in light of its limitations. Importantly, the sample consists of clinically referred adolescents from non-randomly selected treatment sites, which limits generalizability. Nevertheless, it is one of the few studies of justice-involved youth that includes a multi-state sample with consistent use of selected measures across states. Justice-involved youth were aggregated to include both detained youth and youth in the community under supervision by the juvenile court which can obscure potential between-group differences. Yet, even using this broader definition of justice-involved youth we found comparable rates of mental health problems and trauma exposure to previous studies. This provides support for enhancing services for youth with varied levels of involvement in the justice system. Youth in the CDS were clinically referred for trauma treatment, meaning their inclusion in the CDS is predicated on trauma exposure, which contributes to potential overestimation of prevalence rates of trauma exposure. However, it also allowed for the inclusion of a broader range of trauma types and more detailed trauma histories.

Despite these limitations, these findings expand the literature by utilizing a comprehensive trauma history assessment, including a broad range of traumas and age at time of exposure, among a large, multi-state sample. This methodology provides a deeper understanding of justice-involved youth's trauma histories and later mental health problems, which have essential practice and policy implications. Future research should continue to explore developmental pathways from trauma exposure to justice involvement by focusing on the implications of timing of trauma exposure and cumulative exposure across development in order to identify key points for intervention.
